# The impact of HCBS use patterns on older aduts’ physical function among continuous service users

**DOI:** 10.1093/geroni/igab046.3646

**Published:** 2021-12-17

**Authors:** Ya-Mei Chen, Shih-Cyuan Wu, Kuan-Ming Chen, Chen-Wei Hsiang, Shiau-Fang Chao, Ming-Jen Lin, Ji-Lung Hsieh, Yu-Hsuan Chou

**Affiliations:** 1 National Taiwan University, Taipei, Taipei, Taiwan (Republic of China); 2 National Bureau of Economic Research, CAMBRIDGE, Massachusetts, United States; 3 National Taiwan University, National Taiwan University, Taipei, Taiwan (Republic of China); 4 National Taiwan University, Taipei City, Taiwan (Republic of China); 5 Graduate Institute of Journalism, Taipei City, Taipei, Taiwan (Republic of China)

## Abstract

Background Taiwan, one of the fastest aging countries in the world, provides home-and community-based services (HCBS) care recipients through the second 10-Year Long-Term Care Plans (LTC Plans 2.0). The current study aimed to examine the impact of continuous use of HCBS offered by LTC plan 2.0 on older adults’ physical function change overtime. Methods This study used the LTC Plan 2.0 database and the National Health Insurance Plan claim dataset, and included 157,682 clients who had applied for and were evaluated for LTC services for the first time from 2018 through 2020 and continuously used any LTC Plan 2.0 services for six months. Outcome variables were measured by activities ofdaily living (ADLs) and instrumental activities of daily living (IADLs) between baseline assessment and the assessment after the six months of serivce utilization. Latent class analysis and generalized estimating equations were used to investigate the influences of different service use patterns on the changes in physical functions. Results Three subgroups of LTC recipients with different use patterns, including home-based personal care (home-based PC) services (n = 111,976; 71.01%), professional care services (n = 31,584; 20.03%), and community care services (n = 14,122; 8.96%) were identified. When compared to care recipients in the community care group, those in the home-based PC group improved more in IADL scores, and the professional care group improved more ADL scores. Conclusion Those receiving more professional services made more progress in ADL ability, but those in home-based PC made more progress in IADL ability.

